# Synergistic Effects of Insulin-like Growth Factor-1 and Platelet-Derived Growth Factor-BB in Tendon Healing

**DOI:** 10.3390/ijms26094039

**Published:** 2025-04-24

**Authors:** Julia Rieber, Petra Wolint, Gabriella Meier-Bürgisser, Esteban Ongini, Pietro Giovanoli, Maurizio Calcagni, Jess G. Snedeker, Johanna Buschmann

**Affiliations:** 1 Division of Plastic Surgery and Hand Surgery, University Hospital Zurich, Sternwartstrasse 14, 8091 Zurich, Switzerland; julia.rieber@usz.ch (J.R.); petra.wolint@usz.ch (P.W.); gabriella.meierbuergisser@usz.ch (G.M.-B.); pietro.giovanoli@usz.ch (P.G.); maurizio.calcagni@usz.ch (M.C.); 2 Institute for Biomechanics, ETH Zurich, 8092 Zurich, Switzerland; esteban.ongini@hest.ethz.ch (E.O.); jess.snedeker@hest.ethz.ch (J.G.S.); 3 Balgrist University Hospital, University of Zurich, 8008 Zurich, Switzerland

**Keywords:** emulsion electrospinning, Fourier Transform Infrared Spectroscopy (FTIR), Seahorse, gene expression, insulin-like growth factor-1 (IGF-1), platelet-derived growth factor-BB (PDGF-BB) release kinetics, scanning electron microscopy (SEM), chorioallantoic membrane (CAM)

## Abstract

Tendon ruptures are common musculoskeletal injuries associated with prolonged healing and complications such as adhesion formation and rerupture. Despite advancements in treatment strategies, full functional recovery remains a challenge. Growth factors (GFs) like insulin-like growth factor-1 (IGF-1) and platelet-derived growth factor-BB (PDGF-BB) play key roles in tendon repair and may have synergistic effects when applied together. To support tendon healing, a bioactive electrospun polymer scaffold made of Degrapol^®^ (DP) was developed, incorporating IGF-1, PDGF-BB, or both. A range of in vitro and in vivo analyses were performed to assess scaffold structure, cell behavior, gene expression, metabolism, and biomechanical and adhesion outcomes three weeks post-surgery. Interestingly, the combined application of IGF-1 and PDGF-BB did not simply amplify individual effects but showed a complex interaction. Depending on the parameter and time point, the combination led to either enhanced or reduced responses compared to single-factor treatments, indicating a synergistic modulation rather than a purely additive effect. These findings suggest that the combination of IGF-1 and PDGF-BB can modulate key cellular and molecular processes in tendon regeneration, making this approach a promising strategy to improve tendon healing.

## 1. Introduction

Tendon healing is a complex and highly orchestrated biological process that involves overlapping inflammatory, proliferative, and remodeling phases [1,2]. Following injury, platelets and immune cells infiltrate the wound site and release various growth factors (GFs), including platelet-derived growth factor (PDGF) [3], transforming growth factor-beta (TGF-β) [3,4,5], and insulin-like growth factor-1 (IGF-1), which initiate a cascade of cellular responses [5,6,7].

In the subsequent proliferative phase, fibroblasts and tenocytes proliferate and produce extracellular matrix components such as type III collagen (Col3) supported by angiogenic and mitogenic signals from vascular endothelial growth factor (VEGF), fibroblast growth factor (FGF), and others [1,2,7]. The final remodeling phase, which can span months, is characterized by the reorganization of collagen fibers, particularly the transition from Col3 to the mechanically stronger collagen type I (Col1), largely regulated by mechanical loading and cell–matrix interactions [5]. Despite this remodeling, the newly formed tendon tissue often remains biomechanically inferior to uninjured tendon, with some disorganized matrixes and scars persisting [1,2]. In this study, we aim to enhance the healing process, especially in the first two phases, to decrease the risk of rerupture and adhesion formation, common problems in tendon healing [8].

Among the array of GFs involved in tendon regeneration [5,9], IGF-1 and PDGF-BB have emerged as potent modulators due to their potentially synergistic effects on cell proliferation, matrix synthesis, and cell migration [10,11]. PDGF-BB stimulates mesenchymal stem cell (MSC) activation and upregulates IGF-1 expression [11,12], while IGF-1 enhances matrix deposition and promotes cell survival via the PI3K/Akt/mTOR signaling pathway [13,14]. This coordinated activity not only improves tissue organization but also enhances mechanical strength in healing tendons, as demonstrated in several in vivo models. In particular, the Achilles tendon (AT), with its poor vascularization and exposure to high mechanical loads, presents unique challenges in the healing process. Here, IGF-1 and PDGF-BB have shown promising outcomes in improving both cellular activity and biomechanical properties [12,15].

In addition to biological interventions, biomechanical support through implants, scaffolds, prosthetic devices, or allografts is often employed to facilitate tendon healing. These devices provide mechanical stabilization, guide tissue regeneration, and can serve as delivery platforms for GFs or cells [11,16,17]. For example, biodegradable scaffolds embedded with PDGF-BB have been shown to enhance tendon matrix organization and strength while minimizing adhesion formation [14]. Furthermore, recent advances in bioengineered materials have led to the development of smart implants capable of responding to mechanical stimuli or releasing therapeutic agents in a controlled manner [18]. PDGF-BB was delivered over the long term by porous microspheres, demonstrating potential in rat AT healing [19]. Electrospun fibers poly (lactide-co-glycolide) acid (PLGA) with PDGF-AA could stimulate and promote tendon healing [11]. Additionally, the application of rhPDGF-BB has been shown to enhance tendon remodeling and improve biomechanical characteristics in rat models of AT injury. Moreover, combining tendon-derived stem cells (TDSCs) with scaffold materials has exhibited synergistic regenerative effects, significantly supporting tendon repair processes [20,21].

In this study, we aim to build upon previous findings regarding the release of PDGF-BB [22] and IGF-1 [23] from tubular implant materials applied in a rabbit AT full transection model. To this end, we developed and characterized a novel three-layered tubular scaffold. The innermost layer, in direct contact with the tendon, is loaded with PDGF-BB to promote early cellular recruitment and proliferation. This is followed by a middle layer containing IGF-1 to support matrix synthesis and tenocyte activity. The outermost layer is composed of a pure DegraPol^®^ (DP) layer with a smooth surface designed to face the surrounding tissue and minimize adhesion formation.

To further explore the synergistic effects of PDGF-BB and IGF-1, we conducted in vitro analyses using rbTenocytes. This included gene expression profiling, cell morphology assessment, and metabolic characterization via Seahorse analysis. These investigations provide a deeper understanding of how these GFs influence tenocyte behavior at the cellular level. Complementarily, in vivo application of the tubular implant allows evaluation of post-healing biomechanical properties and the extent of adhesion to surrounding tissue. Together, these findings offer valuable insight and may pave the way for the development of clinically relevant tendon repair strategies. In this study, we use the term “synergistic” to describe the interactive effects of IGF-1 and PDGF-BB when applied in combination. Specifically, we refer to synergy as any modification of the response that results from the interaction of the two growth factors—whether enhanced, reduced delayed, or otherwise altered—compared to the response observed with each growth factor alone.

## 2. Results

### 2.1. Charcterization of DP Scaffolds

**Figure 1:**
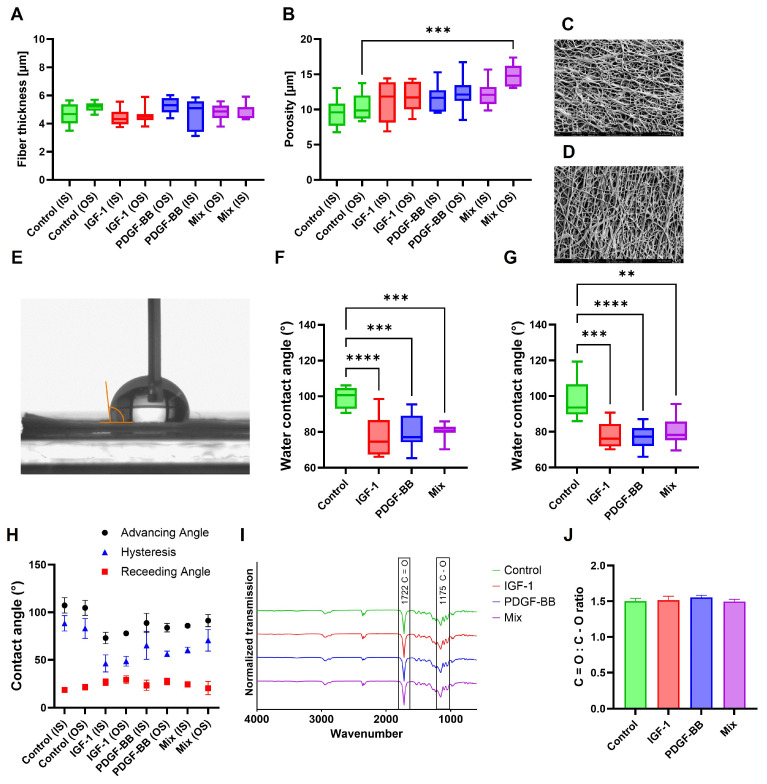
Characterization of electrospun DP meshes containing IGF-1, PDGF-BB, or a mix of both GFs. Fiber thickness (**A**) and porosity (**B**) measured on the IS and OS. SEM images of inner surface of control scaffold (**C**) and outer surface of control scaffold (**D**) representing different surface structures. Representative image of WCA measurement, the orange angle indicating the measured angle (**E**). WCA results of inner surface (**F**) and outer surface (**G**) showing significant differences in hydrophilicity between the groups. Advancing and receding WCAs (**H**), along with hysteresis, for both IS and OS. FTIR spectra of the samples, indicating most significant peaks (**I**). C=O:C–O ratio derived from FTIR analysis (**J**). Data are presented as box-and-whisker plots showing the interquartile range and the full data range, *n* = 9 (**A**,**B**,**F**,**G**). Data are shown as mean and SD (*n* = 3), statistics are shown in Figure S1H and *n* = 9 (**J**). For all, normality was assessed using the Shapiro–Wilk test. As data were normally distributed, group comparisons were performed using one-way ANOVA followed by Tukey’s multiple comparisons test (**A**,**B**,**F**,**G**,**H**); Kruskal–Wallis with Dunn’s multiple comparison was used for comparisons of C=O:C–O (**J**). *p*-values ≤ 0.05 were considered significant and denoted as: *p* ≤ 0.01 (**); *p* ≤ 0.001 (***); *p* ≤ 0.0001 (****).

The structural characterization of the samples, as shown in Figure 1A,B, indicates that fiber thickness remained relatively consistent across all groups, with no significant differences between the inner surface (IS) and outer surface (OS). Porosity measurements show that most groups with GF did not exhibit significant differences compared to the control. However, the OS of the mixed scaffolds showed a slight but statistically significant increase in porosity compared to the control (OS). SEM images of IS (Figure 1C) and OS (Figure 1D) provide a closer look at the fiber structure of the control group. The inner surface (C) appears more compact and densely structured, with fibers sticking together. In contrast, the outer surface (D) exhibits a more open and porous structure, which aligns with the quantitative porosity measurements (Figure 1B) showing slightly higher porosity in the outer surface.

The water contact angle (WCA) analysis of IS (Figure 1F) and OS (Figure 1G) shows that the static WCA was significantly lower in the GFs containing samples compared to the control, indicating increased hydrophilicity. Furthermore, contact angle hysteresis (Figure 1H) followed a similar trend, with the control group having the highest values. The rather high hysteresis is an indication of higher scaffold heterogeneity, which can be confirmed with SEM images. The inner and outer surfaces of GF groups exhibited comparable results, with slight variations in absolute values.

Fourier Transform Infrared Spectroscopy (FTIR) analysis (Figure 1I) indicates that all scaffolds tested exhibited highly similar spectra, suggesting that the overall chemical composition remained unchanged despite the addition of GFs. The C=O:C–O ratio (Figure 1J) was measured to compare the most prominent peaks in the spectra, resulting in no significant difference between GF scaffolds and the control. These results suggest that the overall composition of the fibers remained unchanged.

### 2.2. Release of GF from Scaffolds

**Figure 2:**
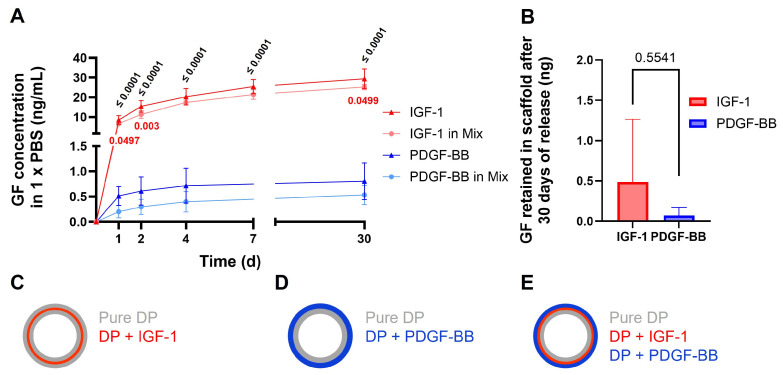
Release of PDGF-BB and IGF-1 from tubular implants. The release of IGF-1 and PDGF-BB was measured from different tubes either containing one of the GFs (dark colors) or both (light colors) in combination (mixed tube as described before). The cumulative release was measured over time on d1, d2, d4, d7, and d30 and displayed on a curve (**A**) where each datapoint represents the mean and SD (IGF-1 and IGF-1 in mix *n* = 4, PDGF-BB *n* = 8, PDGF-BB in mix *n* = 7). Amount of PDGF-BB and IGF-1 was retained in the mixed scaffold after 30 days of release (**B**), obtained by degradation with lipase represented as mean and SD (*n* = 6). Schematical presenting three-layer IGF-1 tube (**C**), two-layer PDGF-BB tube (**D**), and three-layer mixed tube (**E**). Normal distribution was tested with the Shapiro–Wilk test; one-way ANOVA with Tukey’s multiple comparison test was performed to compare groups at individual time point. For significant differences, *p*-value is indicated in black for comparison between IGF-1 groups and PDGF-BB groups and in red for comparison of individual IGF-1 groups.

The release profile of the GFs from the scaffold was governed by a dual mechanism. Initially, the GFs were released through diffusion, as they were emulsified within the DP solution. Over time, as the DP began to degrade, GFs were gradually released, contributing to a sustained delivery profile. This combination of diffusion-controlled and degradation-mediated release ensured a prolonged and controlled availability of the bioactive factors (Figure 2).

The in vitro release kinetics of IGF-1 and PDGF-BB from different scaffolds revealed that the cumulative release of IGF-1 from the scaffold containing IGF-1 alone was significantly higher compared to its release from the mixed scaffold containing both IGF-1 and PDGF-BB on some days. Similarly, PDGF-BB release from the mixed scaffold was lower than from the scaffold containing PDGF-BB alone, but without a significant difference. Furthermore, both IGF-1 release profiles—whether from the IGF-1-only scaffold or the mixed scaffold—were significantly higher than the PDGF-BB release profiles.

The release was especially high on the first day, followed by a slow and steady release up to day 30. The release from the mix tube was smaller compared to the release where each GF was emulsified individually to the tubes, but still there was no significant difference. The amount of GF still present in the scaffold after 30 days of release was extracted by lipase degradation, where the scaffold was hydrolyzed. The amount of IGF-1 was significantly higher (*p* = 0.5541) than the amount of PDGF-BB. The IGF-1 amount per tube piece was estimated to 45 ng, whereas for PDGF-BB, the amount was 59 ng per piece where release was measured.

### 2.3. Gene Expression Analysis

**Figure 3:**
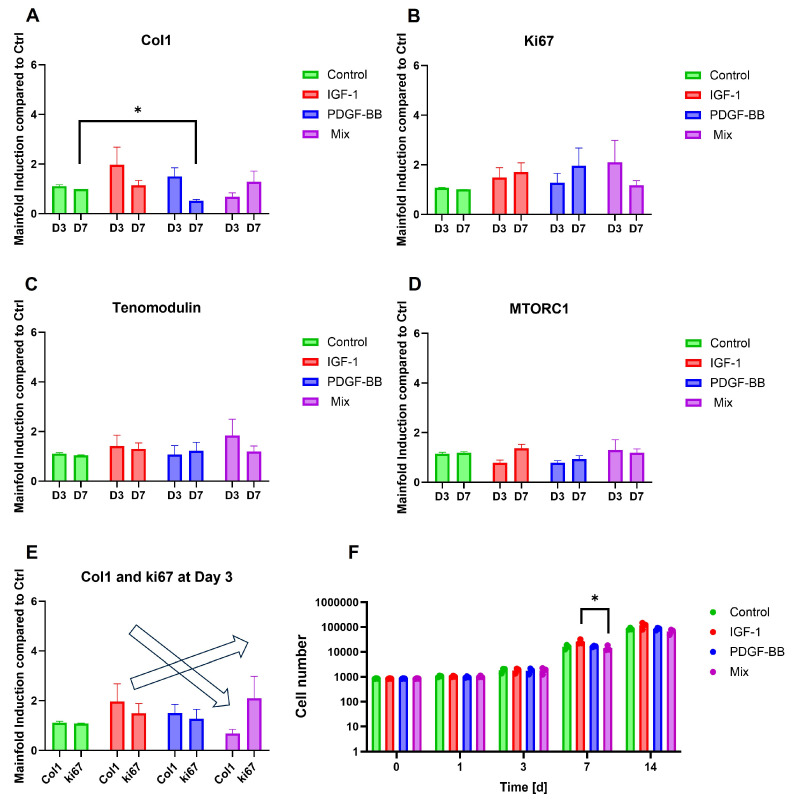
Gene expression analysis and metabolic activity. Data are shown as mean and SEM obtained from three donors of rabbit tenocytes cultured with three different GF treatments (IGF-1 1 ng/mL, PDGF-BB 25 ng/mL, and mix of same concentrations) and a control. Relative gene expression levels of Col1 (**A**), Ki67 (**B**), tenomodulin (**C**), and MTORC1 (**D**) at d3 and d7, expressed as fold induction compared to the control (normal culture medium). Combined analysis of Col1 and Ki67 expression at d3 (**E**), illustrating an inverse trend where Col1 expression decreased while Ki67 increased from IGF-1 to the PDGF-BB treatment and mix. Cell proliferation was measured by alamarBlue™ assay over 14 days (**F**). Data are shown as mean and SD, *n* = 12, where every four technical replicates were averaged. Normality was assessed using the Shapiro–Wilk test. As data were not normally distributed, group comparisons were performed using Kruskal–Wallis with Dunn’s multiple comparison, *n* = 9, where every three technical replicates were averaged. *p*-values ≤ 0.05 were considered significant and denoted as: *p* ≤ 0.05 (*); if no significance is indicated, no significant difference was obtained between groups.

The gene expression analysis revealed distinct trends in response to the different treatments (Figure 3A–D). Col1 expression (Figure 3A) showed a noticeable increase in the IGF-1 group at d3, followed by a reduction in expression at d7 across all groups. Ki67, a proliferation marker (Figure 3B), showed an increase from d3 to d7, particularly in the PDGF-BB group, whereas in the mixed group, the effect was reversed. The inverse relationship between Ki67 and Col1 is shown in Figure 3E, where Col1 expression decreased while Ki67 expression increased from IGF-1 to the PDGF-BB group and was highest in the mixed group.

Expression levels of tenomodulin (Figure 3C), a tendon-associated marker, and MTORC1 (Figure 3D), a central regulator of cellular metabolism, did not exhibit pronounced treatment-dependent variations. Nonetheless, an inverse trend in gene expression dynamics was observed between the individual growth factor treatments (IGF-1 and PDGF-BB) and their combined application (Mixed) across all analyzed genes.

Specifically, for Col1, IGF-1, and PDGF-BB, treatments led to higher expression levels at d3 compared to d7, whereas the Mixed treatment resulted in the opposite pattern, with increased expression at d7. Conversely, for tenomodulin, Ki67, and MTORC1, the mixed treatment induced higher expression at d3 with a subsequent decrease by d7, whereas the individual treatments displayed the opposite trend, with lower expression at d3 and increased levels at d7.

Cell proliferation measured by the alamarBlue™ assay (Figure 3F) demonstrated a steady increase in cell number across all conditions, with a large increase from d3 to d7 first. A significant lower proliferation for the mixed group (lower cell number) compared to the IGF-1 group was detected at d7, whereas at d14, the cell numbers for all groups showed no difference anymore.

### 2.4. Cell Seeding on Scaffolds

**Figure 4:**
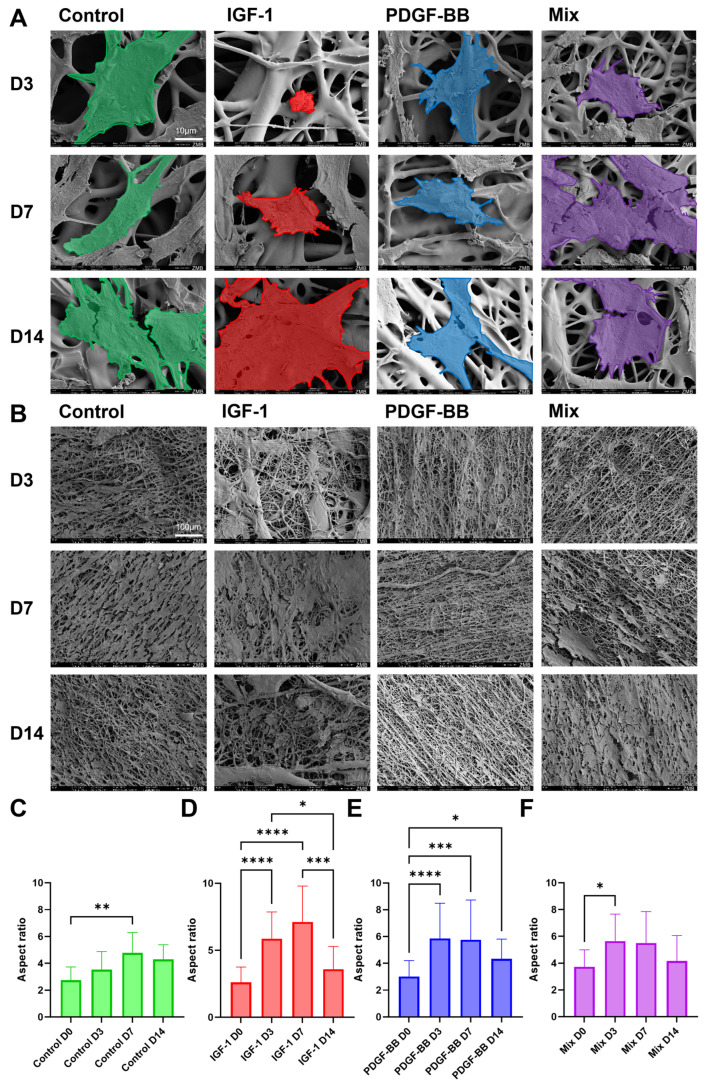
Cell morphology and interaction with scaffolds over time. Representative SEM images (scale bar 10 µm) of cells cultured on different scaffolds (control, IGF-1, PDGF-BB, mixed) at d3, d7, and d14, with cells colored for better visualization (**A**). SEM images of scaffolds (scale bar 10 µm) at d3, d7, and d14 across conditions, with cell coverage over time (**B**). Quantitative analysis of aspect ratio (length/width), measured from cell culture images, for control (**C**), IGF-1 1 ng/mL (**D**), PDGF-BB 25 ng/mL (**E**), and mixed (same concentration of GF) (**F**) over time, showing significant differences in cell morphology between groups. Data are shown as mean and SD. Normality was assessed using the Shapiro–Wilk test. As data were not normally distributed, group comparisons were performed using Kruskal–Wallis with Dunn’s multiple comparison. *p*-values ≤ 0.05 were considered significant and denoted as *p* ≤ 0.05 (*); *p* ≤ 0.01 (**); for *p* ≤ 0.001 (***); for *p* ≤ 0.0001 (****).

Scanning electron microscopy (SEM) analysis was performed to assess cell adhesion, morphology, and interaction with fiber scaffolds over time (Figure 4A). At d3, some differences in cell attachment and spreading were observed among the groups. While the control group seemed more spread out, the cell on the IGF-1 scaffold was noticeably smaller and more rounded. In contrast, PDGF-BB exhibited an elongated morphology with early alignment along the fibers. The mixed group showed moderate spreading and elongation. For the next days, the control cell remained flattened with minimal elongation, showing weak interaction with the scaffold. IGF-1 cells exhibited increased spreading over time but retained a rounded morphology, indicating adhesion without significant elongation. In contrast, PDGF-BB incorporated with the scaffold, displaying strong elongation and alignment along the fibers for the growing cells. The mixed group demonstrated a combination of spreading and elongation.

SEM analysis of scaffold coverage with rabbit tenocytes (Figure 4B) revealed distinct trends across the different treatment groups over time. In the control, IGF-1, and PDGF-BB groups, cell coverage peaked at d7 before decreasing at d14, suggesting an initial phase of strong attachment followed by potential cell detachment or migration. In contrast, in the mixed group, cell coverage gradually increased over time, reaching its highest level at d14, indicating a delayed but sustained cell attachment process. Overall, scaffold coverage was highest in the control and IGF-1 groups, followed by the mixed group, while PDGF-BB showed the least coverage throughout the experiment.

### 2.5. Aspect Ratio

The aspect ratio analysis (Figure 4C–F) quantifies the morphological changes observed during cell culture among the different treatments. Microscopy images (Figure S2) were taken at d0, d3, d7, and d14, and the aspect ratio was calculated (cell length/cell width). In the control group (Figure 4C), a gradual but minor increase in aspect ratio was observed up to d7, suggesting limited cell elongation. In contrast, cells in the IGF-1 group (Figure 4D) and PDGF-BB (Figure 4E) showed a significant increase in aspect ratio by d3 and d7, indicating early cell elongation, which slightly declined by d14. The mixed group (Figure 4F) followed a similar trend but with slightly less pronounced changes.

### 2.6. Metabolic Analysis with Seahorse

**Figure 5:**
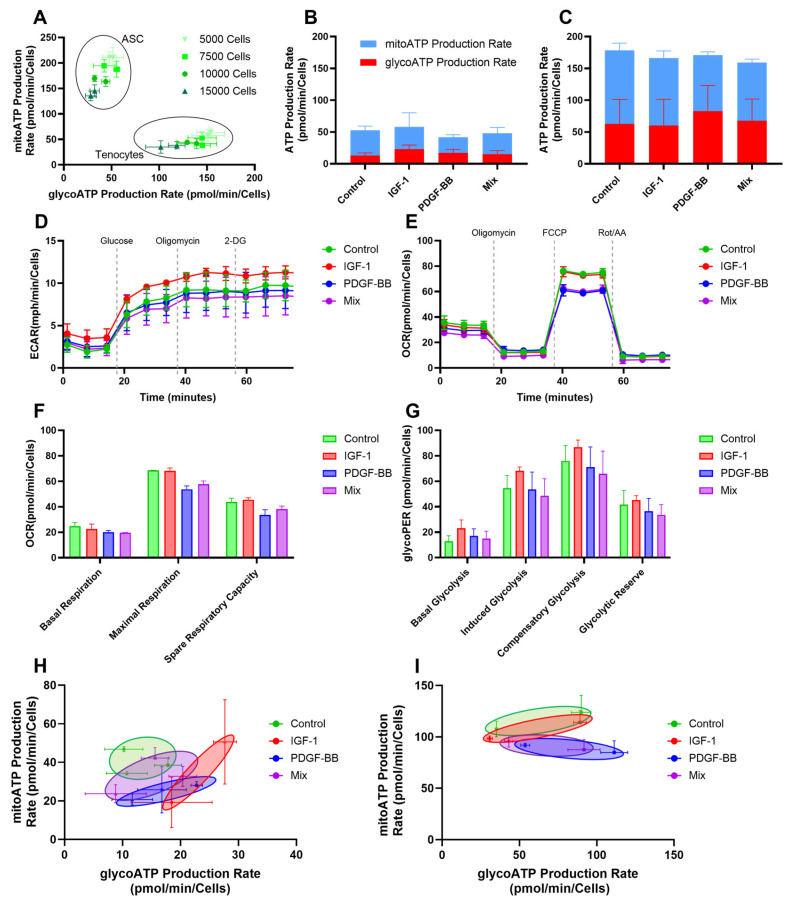
Seahorse metabolic analysis of cells cultured with different GF treatments (control, IGF-1 1 ng/mL, PDGF-BB 25 ng/mL, and mix of same concentrations). Comparison of mitoATP and glycoATP production rates in different cell densities for rbTenocytes and rbASCs (**A**). Total ATP production rate separated into mitoATP and glycoATP production for rbTenocytes (**B**) and rbASCs (**C**), shown as mean and SD. ECAR over time, indicating glycolytic activity in response to metabolic inhibitors, measured with rbTenocytes (**D**). OCR over time (**E**), measured in rbASCs, reflecting mitochondrial respiration, shown as mean and SD. Quantification of basal respiration, maximal respiration, and spare respiratory capacity in rbASCs (**F**). Glycolytic parameters, including basal glycolysis, induced glycolysis, compensatory glycolysis, and glycolytic reserve assessed in rbTenocytes (**G**). Data are shown as mean and SD; no significant differences were detected. For the data representation and statistics, three donors for rbTenocytes and two donors for rbASCs were used, where five technical replicates were averaged. Metabolic phenotyping of cells based on mitoATP vs. glycoATP production, showing metabolic shifts between groups, indicative of a Warburg-like effect. Each data point represents mean and SD of an individual donor per condition for three donors of rbTenocytes (**H**) and two donors of rbASCs (**I**).

Seahorse metabolic analysis revealed distinct metabolic differences between the control and treated groups. Figure 5A showed that rabbit adipose derived stem cells (rbASCs) exhibited higher mitochondrial ATP (mitoATP) production compared to rbTenocytes, which relied more on glycolysis, confirming metabolic differences between cell types.

ATP production rates (Figure 5B,C) indicated that control cells primarily relied on mitochondrial respiration, with a small contribution from glycolysis. In contrast, treatment with IGF-1 exhibited an increase in glycolytic ATP production in rbTenocytes, while PDGF-BB treatment showed a shift toward glycolysis in rbASCs. For both cell types and all conditions, mitoATP remained a dominant contributor.

Extracellular acidification rate (ECAR) showed an increase over time upon glucose addition (Figure 5D), confirming active glycolysis in all groups. The IGF-1-treated group exhibited slightly higher ECAR compared to the control, suggesting an increased reliance on glycolysis. Oxygen consumption rate OCR measurements (Figure 5E) revealed similar baseline respiration across groups, but maximal respiration after FCCP injection showed a slight decrease in the PDGF-BB and mixed groups, whereas IGF-1 exhibited similar values as the control.

Mitochondrial respiration (Figure 5F) and glycolytic activity (Figure 5G) both showed a treatment-dependent metabolic response, though with slight differences in hierarchy. In Panel F, which measures basal respiration, maximal respiration, and spare respiratory capacity in rbASCs, IGF-1-treated cells consistently exhibited the highest values, followed by the mixed group, while PDGF-BB-treated cells remained lower. This suggests that IGF-1 enhances mitochondrial activity. For basal glycolysis, induced glycolysis, compensatory glycolysis, and glycolytic reserve measured in rbTenocytes (Figure 5G), IGF-1-treated cells again showed the highest glycolytic activity, followed by PDGF-BB, which was higher than the mixed treatment. This suggests that while IGF-1 drives the strongest metabolic activation across both mitochondrial and glycolytic pathways in different cell types, PDGF-BB preferentially enhances glycolysis in rbTenocytes, whereas the mix maintains an intermediate effect in mitochondrial respiration in rbASCs but is less effective in activating glycolysis in rbTenocytes.

The energy plot of donors presented individually under each condition for three donors of rbTenocytes (Figure 5H) and two donors of rbASCs (Figure 5I) highlights a shift, where the GF treated group showed increased glycolytic ATP (gylcoATP) production with a relative decrease in mitoATP production, known as the Warburg shift.

### 2.7. CAM Assay

**Figure 6:**
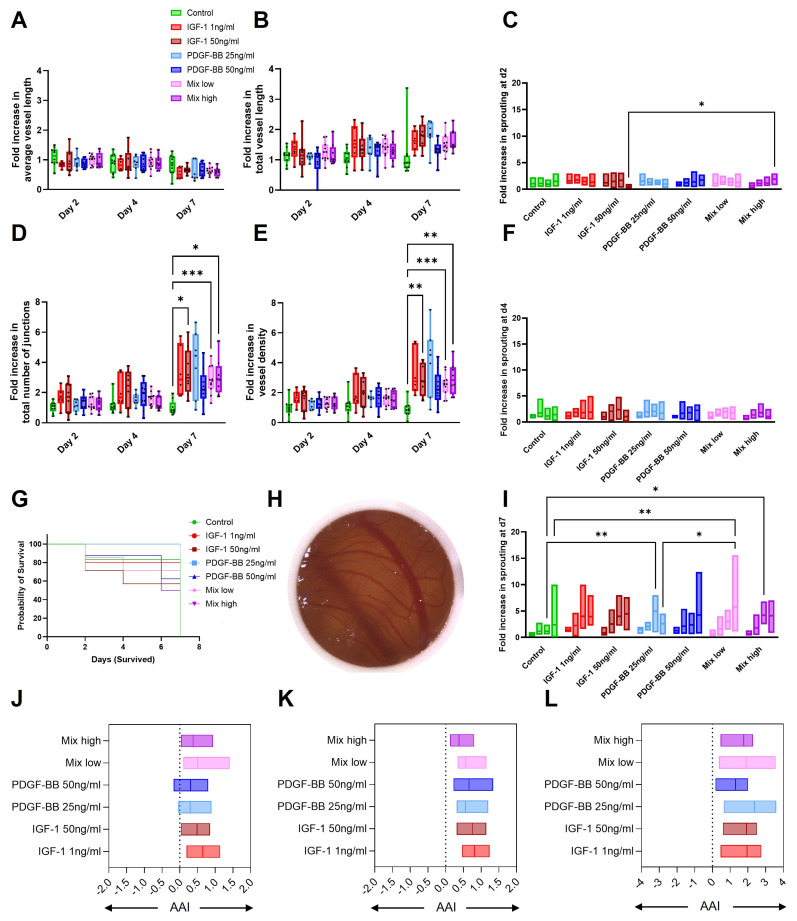
Quantification of angiogenesis parameters in the CAM assay for different treatment groups over time (d2, d4, and d7). Data are expressed as fold increase compared to d0 and shown as box-and-whisker plots showing the interquartile range and the full data range with individual values. Average vessel length (**A**), total vessel length (**B**), total number of junctions (**D**), vessel density (**E**). Normal distribution was tested with the Shapiro–Wilk test; two-way ANOVA with Tukey’s multiple comparison test was performed to compare time points and treatment (*n* = 6–11). Vessel hierarchy at d2, d4, and d7 includes primary, secondary, tertiary, and quaternary sprouts, shown as floating bars with a full data range (min to max) in this order from left to right for each group (**C**,**F**,**I**). The CAM was treated for 7 days with different GF solutions, and survival is shown as a survival plot (**G**). A representative image of the CAM on d4 assessed trough microscopy (**H**). The Angiogenic Activity Index (AAI) was calculated for d2 (**J**), d4 (**K**), and d7 (**L**) based on the parameters junctions (**D**), vessel hierarchy (**C**,**F**,**I**), total vessel length (**B**), and vessel density (**E**) for each timepoint. *p*-values ≤ 0.05 were considered significant and are denoted as *p* ≤ 0.05 (*); *p* ≤ 0.01 (**); *p* ≤ 0.001 (***).

For all groups, the survival of the chicken embryos was comparable with no significant differences (Figure 6G). As a starting point, the vascularization of the CAM had a different extent, and the parameters were all calculated in fold increases to d0. The CAM assay was used to evaluate angiogenic progression via angiogenic parameters, including average vessel length (Figure 6A), total vessel length (Figure 6B), number of junctions (Figure 6D), and vessel density (Figure 6E), to assess the extent and complexity of vascularization. Across most of the parameters, angiogenic measurements increased over time, with the most significant changes observed at d7.

All GF treatments induced an angiogenic response, increasing vessel density and vascular junctions to different extents. Also, for total vessel length, a trend of increased values for GF treatment groups can be visualized. In contrast, the average vessel length (Figure 6A) remained the same over time and among all groups with a decrease on d7. Vessel hierarchy changed progressively over time. On d2 (Figure 6C), no differences were observed in the number of primary, secondary, tertiary, or quaternary vessels. However, by d4 (Figure 6F), a shift became apparent, with the number of smaller vessels (tertiary and quaternary) beginning to exceed that of the larger primary and secondary vessels. This trend became even more pronounced by d7 (Figure 6I), indicating a progressive increase in vascular branching and micro vessel formation over time. Importantly, no differences in vessel numbers or distribution patterns were observed between the treatment groups and the control, suggesting that the observed vascular changes reflect a time-dependent remodeling process rather than a treatment-specific effect.

PDGF-BB, especially in the lower concentration, displayed a response, promoting vessel elongation, branching, and vessel complexity. A similar trend was observed for the other GF groups, whereas for the mixed group, the higher concentration seemed to improve angiogenesis more than the lower concentration.

IGF-1 enhanced vessel elongation and branching and was slightly more efficient at earlier time points compared to the other GF treatments. The control group consistently displayed the lowest angiogenic activity, reinforcing the necessity of treatment-induced stimulation for vascular network formation, but the average vessel length was highest in the control, indicating smaller development of smaller vessels.

The CAM assay results indicate that angiogenesis evolved dynamically across treatment groups. Figure 6A,B shows that average vessel length and total vessel length remained relatively stable across groups at d2 and d4, but by d7, IGF-1 50 ng/mL and PDGF-BB 25 ng/mL exhibited an increase in total vessel length compared to the control.

A more pronounced effect is observed in Figure 6D,E, where the total number of junctions and vessel density show notable differences by d7. Low Mixed groups demonstrated the most significant increases in total number of junction formations and vessel density (*p* < 0.001), closely followed by IGF-1 1 ng/mL and high Mixed groups, suggesting that these conditions promote a higher degree of vascular complexity.

To assess angiogenic potential, the Angiogenic Activity Index (AAI) [24] was calculated based on junction count, total vessel length, vessel density, and vessel hierarchy. Using the control group as a baseline for physiological vascularization, the angiogenic response of six different treatment groups was evaluated on different time points (Figure 6J–L). All treatment groups exhibited a pronounced angiogenic activity compared to the control. Moreover, the angiogenic effect increased progressively over time, becoming more evident from d2 to d4 and reaching its peak at d7.

### 2.8. In Vivo Mechanical Testing

**Figure 7:**
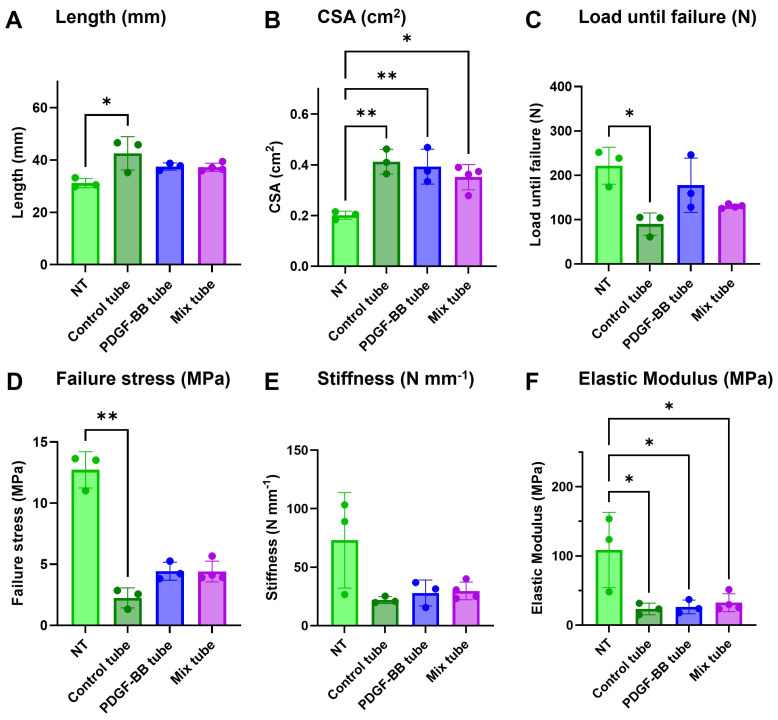
Biomechanical properties of the AT at 3 weeks post-operation with application of different tubular implants. Tendon length (**A**), cross-sectional area (CSA) (**B**), load until failure (**C**), failure stress (**D**), stiffness (**E**), and elastic modulus (**F**) were measured for non-treated tendons, *n* = 3 (NT) and Achilles tendon transection treated with a control tube, *n* = 3 (no GF), PDGF-BB tube (*n* =3), or Mix tube (*n* = 4). Data are shown as mean and SD with individual data points. Normal distribution was tested with Shapiro–Wilk test, one-way ANOVA with Tukey’s multiple comparison test to compare groups (**A**,**B**,**E**,**F**), or Kruskal–Wallis with Dunn’s multiple comparison (**C**,**D**). *p*-values ≤ 0.05 were considered significant and are denoted as *p* ≤ 0.05 (*); *p* ≤ 0.01 (**).

Biomechanical testing revealed significant differences in tendon properties across treatment groups (Figure 7) after 3 weeks in vivo. Tendon length (Figure 7A) was significantly shorter in the control tube compared to non-treated tendons and GF tube groups (*p* < 0.05). NT cross-sectional area (CSA, Figure 7B) was significantly larger in the GF groups compared to the control tube, indicating potential tissue remodeling or scar formation.

Load until failure (Figure 7C) was highest in the non-treated tendons (NT) and significantly reduced in the control tube, while the PDGF-BB tube showed the highest load until failure among the groups with tube application. Failure stress (Figure 7D), stiffness (Figure 7E), and elastic modulus (Figure 7F) followed a similar pattern, with the non-treated tendons exhibiting the highest values, while all treatment groups showed reduced mechanical strength. Notably, the PDGF-BB and Mix tube groups consistently outperformed the control tube group across several parameters, suggesting a more favorable mechanical outcome.

### 2.9. Adhesion

**Figure 8:**
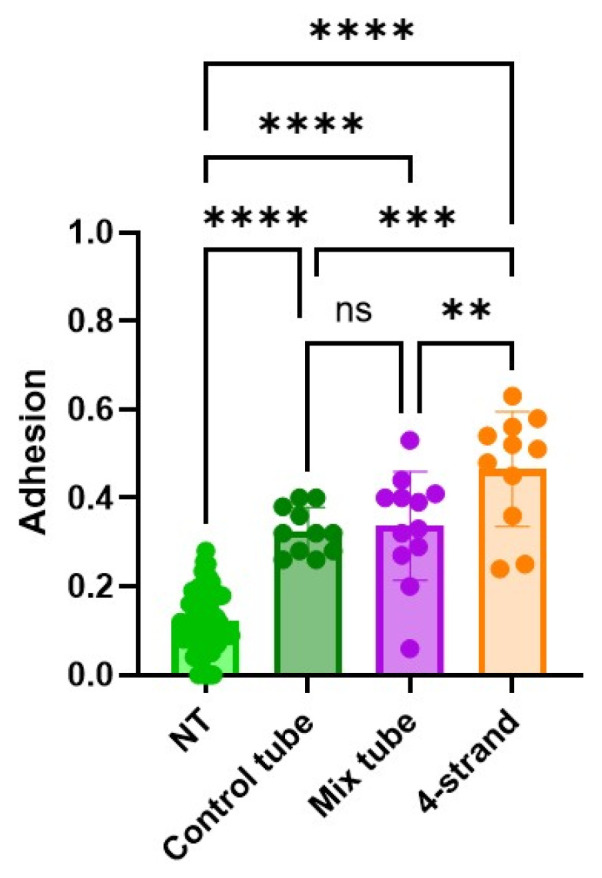
Quantification of adhesion content in extracted rbAT 3 weeks post-surgery. Adhesion quantified and shown as fraction for four groups non treated (*n* = 61), four-strand suture with control tube application (*n* = 11), 4-strand suture with Mix tube application (*n* = 12), and 4-strand suture only (*n* = 11). Data are shown as mean and SD with individual data points. Normal distribution was tested with Shapiro–Wilk test; one-way ANOVA with Tukey’s multiple comparison test was performed to compare groups. *p*-values ≤ 0.05 were considered significant and are denoted as *p* ≤ 0.01 (**); *p* ≤ 0.001 (***); *p* ≤ 0.0001 (****); ns = not significant.

Adhesion extent was assessed for four different conditions (not treated = NT, control tube (pure DP tube), Mix tube (both GFs), and 4-strand Becker suture without a tube). In the NT tendon, the adhesion was significantly the lowest among all groups (Figure 8). As expected, the tendons where a tube was applied had significantly less adhesion formation compared to the suture alone.

## 3. Discussion

Tendon healing is a multi-step process involving different healing steps where analyzing structural integrity, cellular dynamics, metabolic activity, angiogenesis, and biomechanical properties is important [7,25,26].

When addressing tendon healing, two major challenges [27,28] arise during the recovery process. The first issue is the high risk of tendon rerupture [29], which often occurs when patients apply an excessive load too early. At this stage, the tendon has not yet regained sufficient strength to withstand mechanical forces, making it vulnerable to failure. On the other hand, prolonged immobilization can lead to adhesion formation [30] between the healing tendon and surrounding tissues, ultimately reducing function and flexibility.

These adhesions primarily develop during the proliferation phase, a critical stage characterized by extensive cell proliferation and deposition of Col3. This phase plays a key role in tissue repair but also represents a period of mechanical weakness, increasing the risk of rerupture. Since the tendon has not yet regained its original structural integrity, careful management of loading and immobilization are crucial to achieving optimal healing outcomes.

For this purpose, implant material or prosthetic devices which can be applied easily during surgery are of big interest. The DP tubes, which are applied over a conventionally sutured tendon, are not only biodegradable but are also elastic so they fit different tendon sizes and can be loaded with different GFs or other biomolecules. Depending on the composition of the different layers, the tube can further enhance the anti-adhesive effect by adding hyaluronic acid [31] or significantly increase tendon biomechanics after three weeks in vivo by adding a layer of PDGF-BB [32]. By combining the effect of PDGF-BB [22] and IGF-1 [23] in one tube, we aimed to further enhance tendon healing by inducing a synergistic effect of these GFs [33].

IGF-1 on its own is known to enhance DNA and protein synthesis, mainly the production of Col1 [34,35], and in vitro it stimulates tenocyte proliferation [36]. Similarly, PDGF-BB is known for its potent mitogenic and chemotactic effects on mesenchymal cells, including tenocytes, and plays a crucial role in tissue repair by stimulating cell proliferation, migration, and extracellular matrix production [3,35,37,38,39]. Their combined application has been shown to have a synergistic effect on tendon-associated cells. Studies have reported that the combination of 100 ng/mL IGF-1 with 50 ng/mL PDGF-BB resulted in the highest proliferation levels in dose–response experiments using tenocyte in vitro cultures [33]. Furthermore, a combination of 100 ng/mL IGF-1, 10 ng/mL basic Fibroblast Growth Factor (bFGF), and 100 ng/mL PDGF-BB has been demonstrated to synergistically enhance the proliferation of adipose-derived stem cells, which were intended for repopulating a hydrogel designed for tendon repair [34]. These findings highlight the importance of GF interactions in optimizing cell responses for tendon regeneration, suggesting that a multifactorial approach may be more effective than the application of single factors alone.

Given the significant size difference between IGF-1 (7.6 kDa) [40] and PDGF-BB (24 kDa) [14], our previous emulsion electrospinning protocols [22] had to be adapted to optimize the controlled release of both GFs. Initially, we fabricated a two-layered DP tube containing IGF-1, analogous to the approach previously used for PDGF-BB (Figure 2D) [22]. However, this design led to a rapid burst release of IGF-1 within the first hour [41], indicating suboptimal release kinetics. To address this, we developed a three-layered tube structure [23] to delay the release of IGF-1 (Figure 2C). For this approach, the PDGF-BB is incorporated into the third layer (Figure 2C). The innermost layer consists of pure DP, providing structural integrity, while the middle layer contains IGF-1, incorporated via emulsion electrospinning using a water-in-oil emulsion with aqueous IGF-1 dispersed in DP. The outermost layer contains PDGF-BB emulsified the same way, allowing for a controlled release over time.

This layered configuration increases the diffusion distance for IGF-1, resulting in a more sustained release profile over several days (Figure 2A). Additionally, the outer PDGF-BB layer enables the larger molecule to diffuse in a fast and sustained manner. This approach ensures a controlled, time-dependent, and simultaneous delivery of both GFs, circumventing natural GF dynamics, where PDGF-BB induction appears earlier than IGF-1 [42]. Important for the in vivo application is to flip the tubes inside out, so that the PDGF-BB layer is in direct contact with the tendon and the pure DP side is facing the outer tissue.

In SEM analysis, the inner surface of the tubes could be characterized to be smoother and more compact, with smaller pores, whereas the outer surface is more open, rough, and exhibits a higher variance in pore size (Figure 1B). These findings are in accordance with earlier studies, where DP tubes with different compositions were characterized [22,23,31]. These observations suggest that even before treatment, structural differences exist between the inner and outer surfaces of the fibers, which is expected as the inner surface attaches to the metal rod during electrospinning and is pressed against the collector to be flattened out to a small extent. However, SEM imaging revealed no significant differences in fiber diameters when comparing the inner and outer surfaces, with control and GF-containing fibers exhibiting an average diameter of approximately 5 μm. This fiber thickness is consistent with previously reported values for DP electrospun tubes [23] and other electrospun polymer meshes [31,43]. The large standard deviation observed is indicative of the high variability in fiber thickness. This variability is also reflected in the dynamic WCA (Figure 1G) measurements, where all surfaces exhibited similarly large hysteresis, indicative of a heterogenous surface [44].

Further characterization with WCA measurements [45] indicated that both the inner and outer surfaces of the GF tubes were hydrophilic (Figure 1E,F), with negligible differences between them. This suggests that the small variations in fiber and pore size minimally impact hydrophilicity, implying that the material’s properties play a more significant role. The increased hydrophilicity of the GF tubes compared to the control tubes may be attributed to the incorporation of GF in an aqueous solution during scaffold fabrication, whereas no water droplets are introduced in the control process. These findings align with previous studies demonstrating that scaffold composition and processing conditions significantly influence hydrophilicity and, consequently, cell behavior [46,47]. For instance, research has shown that scaffold morphology and structure can affect hydrophilicity, thereby influencing protein adsorption and cell–material interactions [48].

DP is a block co-polymer composed of polyester urethane units, originally developed for bone tissue engineering [49]. FTIR analysis confirmed the characteristic C=O double bond at 1720 cm^−1^ in both pure and emulsion electrospun tubes, typically associated with polyesters and polyurethanes ranging from 1730–1690 cm^−1^ [50]. Furthermore, all tubes exhibited a very similar fingerprint region in the 1250–900 cm^−1^ range, with a distinct peak at 1150 cm^−1^, characteristic of C–O single bonds. These findings indicate that the overall chemical composition remained unchanged despite the incorporation of GFs. The observed minimal differences were likely due to slight variations in surface interactions rather than significant chemical modifications. This suggests that the integration of GFs does not substantially alter the scaffold’s chemical structure, which is crucial for maintaining its inherent properties. These findings are consistent with previous studies observing that the incorporation of GFs or nanoparticles into scaffolds can be achieved without significantly altering their overall chemical composition, as confirmed by FTIR analysis [51,52]. This preservation of chemical structure is essential for maintaining the desired properties of the scaffold while introducing bioactive functionalities.

The in vitro release kinetics of IGF-1 and PDGF-BB from different tubes were evaluated. In our previous study, we showed a slow and steady release of IGF-1 from such scaffolds, which was in line with our recent findings of the release kinetics [23]. Also, model biomolecules that are non-bioactive, including a low molecular weight compound (fluorescein, 376.27 g/mol) and a high molecular weight compound (FITC-labeled bovine serum albumin, FITC-BSA, 66 kDa), were shown to release in the same profile over time [17].

For both GFs in both tube compositions, the release was the highest on the first day, followed by further release over a time up to 30 days. By degrading the scaffolds with Lipase [32], the GF retained in the scaffold could be measured. From these findings, we can conclude that the release of GFs could possibly be maintained for an even longer time. To ensure that the GFs do not degrade, RSA was added to stabilize them over a longer period [53,54]. In previous research, we also showed that IGF-1 released from DP scaffolds was still bioactive, as it showed the same effect on gene expression of Ki67 and tenomodulin on rbTenocytes [23]. It should be mentioned here as well that the in vivo release will probably be different, as the condition at the injury site varies from current experimental conditions. Due to the lipase degradation of the scaffold, the amount of GF still in the scaffold could be determined for IGF-1 (0.48 ng ± 0.44, 1.1%) and PDGF-BB (0.07 ng ± 0.1, 0.12%) (Figure 2B), which was a smaller amount than already released after 30 days (IGF-1 (26.82 ng ± 2.98, 61%), PDGF-BB (0.53 ng ± 0.19, 0.9%)), indicating that the largest amount of GF especially for IGF-1 is released in the first day, but due to the steady release afterwards, the levels of GF at the wound site can be maintained. Both levels of IGF-1 and PDGF-BB are similar as previously reported [22,23]. Overall the incorporation of IGF-1 seems to be more functional than that of PDGF-BB, for which a large amount seems to be lost or degraded; nevertheless, the functionality of PDGF-BB with such a release profile was already shown in vivo in a rabbit AT [32].

Interestingly, the mixed tube, where IGF-1 and PDGF-BB release was measured from the same tube, had a lower amount of release compared to the tubes where only one GF was contained. This could be explained by the surface structure of the scaffolds, which were slightly altered for the mixed tubes, especially the larger pore size on the OS (Figure 1B). Additionally, the release of IGF-1 was significantly higher than the release of PDGF-BB, which could be attributed to the layering of the tube. As IGF-1 was incorporated into the middle layer and PDGF-BB to the outer, PDGF-BB was more exposed. Due to the addition of ethanol, to remove the polyethylene glycol (PEG) layer and to detach the tube from the rod, it is possible that also parts of the PDGF-BB were washed away and consequently did not remain in the scaffold. Another option to explain the reduced release of PDGF-BB could be the molecular weight differences, as IGF-1 with 7.6 kDa [40] was much smaller than PDGF-BB with 24 kDa [14]. This size difference could delay PDGF-BB, as it needs more time to be released from the pores. However, as the total amount of GF released and retained in the scaffold was significantly higher for IGF-1, the approach of washing away seems more plausible. This is also reflected by the burst release on the first time point, which indicates that the GFs are easily released from the scaffold.

Gene expression analysis highlights key molecular changes during tendon healing. Increased Col1 expression suggests enhanced extracellular matrix synthesis, which is crucial for restoring tendon strength and function [55]. Simultaneously, higher Ki67 levels indicate active cell proliferation, reflecting a regenerative response necessary for tissue repair [56,57]. The upregulation of tenomodulin implies improved tenocyte differentiation and matrix organization, aligning with studies emphasizing its role in tendon maturation and scar prevention [58]. Additionally, increased mTORC1 activity supports anabolic signaling, promoting protein synthesis and cellular growth, essential for tendon remodeling [59,60].

The inverse relationship observed between Ki67 and Col1 expression in Figure 3E—where Col1 expression decreased while Ki67 expression increased from IGF-1 to the PDGF-BB treatment, with the highest Ki67 levels in the mixed group, suggests a synergistic role of IGF-1 and PDGF-BB in tendon healing.

IGF-1 is known to stimulate collagen synthesis, particularly Col1, which is the main component of tendon tissue. Studies have demonstrated that IGF-1 enhances tendon healing by supporting cell proliferation, DNA synthesis, and matrix production, especially Col1 [61]. In equine tenocytes [62], 10 and 100 ng/mL IGF-1 showed an increase in Col1 expression, whereas in rat tail tenocytes [63], this was not demonstrated. In previous studies with IGF-1 on rbTenocytes, 1 ng/mL IGF-1 had the greatest effect on Col1 gene expression on day three [23]. This aligns with the elevated Col1 expression observed in the IGF-1 group, indicating that IGF-1 facilitates early extracellular matrix formation during tendon repair but also cell proliferation.

PDGF-BB is recognized for its potent mitogenic and angiogenic properties, which can accelerate tendon healing [14], but also for increasing tendon cell proliferation and collagen synthesis [3]. In vitro studies have shown that PDGF-BB promotes tenocyte proliferation, leading to increased tenocyte density in tissue [39]. This corresponds with the Ki67 and Col1 expression observed in the PDGF-BB group compared to the control, especially at early timepoints [38].

Studies have explored the combined effects of IGF-1 and PDGF-BB on tendon healing. For instance, a study demonstrated that tendon cells require both PDGF-BB and IGF-1, in addition to mechanical load, to effectively stimulate DNA synthesis [64]. Another study investigated the synergistic effects of GF combinations, including IGF-1 and PDGF-BB, on the repopulation of tendon hydrogel scaffolds by ASCs. The findings suggested that combining these GFs improved cellular proliferation of ASCs seeded onto a tendon extracellular matrix gel [65]. This is in accordance with our findings that in the mixed group, especially on d3, the Ki67 gene expression increased compared to the other groups, whereas Col1 gene expression increased only on d7. On d7 in the alamarBlue™ proliferation assay, the decrease in cell proliferation could also be seen for the mixed groups, which is also reflected in the gene expression data.

In summary, the inverse relationship between Ki67 and Col1 expression across the treatment groups underscores the complementary roles of IGF-1 and PDGF-BB in tendon healing. IGF-1 primarily promotes early collagen deposition, enhancing the structural framework of the tendon, while PDGF-BB stimulates cellular proliferation, replenishing the cellular components necessary for effective repair. Strategically modulating these GFs could lead to improved therapeutic approaches for tendon injuries.

For all different groups, the aspect ratio (Figure 4C–F) increased for d3 and d7 and decreased afterwards for d14 with significant differences. The IGF-1 group exhibited the highest aspect ratio, reaching nearly 10, closely followed by PDGF-BB, indicating significantly elongated cells. In contrast, the control group only reached a value of 6, suggesting less elongation. In the mixed group, the increase in elongation on d3 and d7 was not as high as in the other GF groups. A previous study showed that supplementation of rbTenocytes with 20 ng/mL PDGF-BB slightly decreased the aspect ratio compared to the control on d3 but still had similar values compared to these results [66]. Our results are in accordance with a previous study where different concentrations of IGF-1 were applied to rbTenocytes; especially for 1 ng/mL of IGF-1, the aspect ratio on d3 was increased compared to the control [23]. In our study, we observed that individual IGF-1 and PDGF-BB addition led to significant changes in the aspect ratio of cells over time, suggesting a dynamic response in cell morphology. However, for the mixed group, this increase was not highly expressed. The same trend was also identified when looking at the SEM images acquired after cell seeding on the scaffolds. While the surface coverage of the cells seemed to increase up to d7, it declined on d14. Interestingly, in the mixed group, where the cell surface coverage increased up to 14 days, the pore size was also the highest, which favors cell adhesion in combination with the hydrophilic scaffold surface. Additionally, the heterogeneity of our scaffold led to an irregular distribution of cells [47].

Metabolic reprogramming is a hallmark of cellular adaptation to various treatments and environmental conditions. In this study, we employed Seahorse XF technology to analyze the bioenergetic profiles of cells subjected to different treatments (control, 1 ng/mL IGF-1, 25 ng/mL PDGF-BB, and a mix of these GFs in the same concentration) focusing on ATP production, OCR, ECAR, and the potential induction of a Warburg-like effect. As visualized in Figure 5A, rbTenocytes had a lower metabolic activity in general, being more quiescent, while the rbASCs generally tended to have more oxidative activity. This is in accordance with the fact that tenocytes do not have a high metabolism due to the tendon structure [67], whereas ASCs are known to have a higher metabolism in the range of 50–120 pmol/min/cells under basal conditions [68]. Due to these findings, rbTenocytes underwent a glycolysis stress test, revealing that IGF-1 enhanced both basal conditions and induced glycolysis compared to controls (Figure 5G). This suggests that IGF-1 stimulates glycolytic pathways under normal and stressed conditions. In contrast, PDGF-BB treatment resulted in no difference in induced or compensatory glycolysis, while the mixed group did not exhibit an additive effect, indicating potential pathway interference when both factors were present.

For ASCs, a mito stress test was performed to assess mitochondrial respiration. The control group displayed the highest basal respiration, indicating a reliance on oxidative phosphorylation for energy production. IGF-1-treated cells showed slightly reduced basal respiration but demonstrated the highest maximal respiration upon uncoupler addition, suggesting an enhanced mitochondrial capacity for ATP production. Conversely, PDGF-BB and mixed treatments led to reduced basal and maximal respiration, implying diminished mitochondrial activity and a possible metabolic shift towards glycolysis. Notably, the mixed group results were in between the IGF-1 and PDGF-BB group.

These findings align with existing literature. IGF-1 is known to activate the PI3K/Akt signaling pathway, promoting glycolysis and enhancing mitochondrial biogenesis and function [69,70,71,72,73].

PDGF-BB activates the PI3K/Akt signaling pathway, leading to various cellular responses such as proliferation, migration, and angiogenesis [74]. However, the specific downstream effects of PDGF-BB can differ depending on the cell type and context, resulting in varied metabolic outcomes. For instance, in endothelial progenitor cells, PDGF-BB-induced activation of the PI3K/Akt pathway enhances proliferation, migration, and angiogenesis [74] and influences shifts in energy metabolism [75].

The observed synergy in the combined IGF-1 and PDGF-BB treatment may stem from signaling crosstalk and feedback mechanisms that modulate metabolic responses when both GFs are present simultaneously. Research has demonstrated that high concentrations of IGF-1 can decrease levels of its own receptor (IGF-IR) and insulin receptor substrate-1, potentially altering downstream signaling dynamics [76]. Additionally, integrins have been shown to associate with GF receptors, including the IGF-1 receptor, influencing signaling outcomes [77]. These interactions suggest that the simultaneous presence of IGF-1 and PDGF-BB could lead to complex signaling interplay influencing metabolic effects.

Further, our findings revealed that control cells in both cell types predominantly relied on mitochondrial oxidative phosphorylation (Figure 5B) for ATP production, as evidenced by higher mitoATP levels and lower glycolytic activity. This metabolic phenotype is characteristic of cells with efficient mitochondrial function, ensuring sustained energy production and cellular homeostasis [78].

In contrast, rbTenocytes treated with IGF-1 exhibited a shift toward glycolysis, characterized by increased glycoATP production and reduced mitochondrial respiration; for rbASCs, this shift was more prominent in the PDGF-BB group. This metabolic reprogramming mirrors the Warburg effect [79], a phenomenon where cells preferentially utilize glycolysis for energy production, even in the presence of adequate oxygen levels, as it is faster. Such a shift is often observed in rapidly proliferating cells and is associated with increased glucose uptake and lactate production [80]. These findings are also reflected in the proliferating assay (Figure 3F), where IGF-1 proliferated the most, especially on d7, compared to the other groups, and the mixed group showed the lowest activation of proliferation.

The mixed treatment group displayed a more balanced energy production profile, maintaining a harmony between oxidative phosphorylation and glycolysis. This equilibrium suggests a metabolic flexibility that allows cells to adapt to varying energy demands and environmental conditions [79].

The observed decrease in maximal respiratory capacity in PDGF-BB and mixed group indicates a potential reduction in mitochondrial efficiency or an adaptive response to an altered microenvironment. This aligns with the concept that under certain stress conditions or rapid proliferation, cells may downregulate mitochondrial respiration in favor of glycolytic pathways to meet their energy and biosynthetic precursor demands [81].

The CAM assay is a widely utilized in vivo model for studying angiogenesis—the formation of new blood vessels from existing vasculature. Its accessibility, rich vascular network, and immunodeficient environment make it particularly suitable for evaluating the angiogenic potential of various substances, including growth factors and biomaterials [82,83,84].

Several studies have shown that incorporating growth factors such as VEGF and PDGF into the CAM assay significantly enhances angiogenesis. VEGF increases vessel density and length by stimulating endothelial cell proliferation and migration [83]. PDGF-BB, on the other hand, promotes vessel maturation by recruiting pericytes and smooth muscle cells, contributing to vascular stability [83,85,86]. Similarly, IGF-1 has been observed to promote endothelial cell migration and tube formation, essential steps in the angiogenic process. These effects are mediated through the activation of the PI3K/Akt signaling pathway, which enhances the expression of angiogenesis-related genes and proteins [87,88]. Furthermore, IGF-1 has been shown to reduce inflammation, enhance vascular regeneration, and improve re-epithelialization and collagen deposition in acute wounds, indicating its therapeutic potential in tissue repair and regeneration [89]. Also, in other approaches assessing angiogenesis, IGF-1 and PDGF-BB promoted micro vessel development [90,91]. Thes studies confirm our findings, where all GF groups could increase the AAI compared to the control. The GF, especially of both the mixed and IGF-1 50ng/mL groups, induced a larger number of junctions and a higher vessel density, indicative of more complex vessel formation over time.

The application of PDGF-BB and IGF-1 may have a combinatorial effect on angiogenesis, as both stimulated vessel growth in a rat aortic assay [90]. In a study with platelet rich plasma (PRP), it was shown that the application of PRP led to an increased concentration of GF, including IGF-1 and PDGF-Bb, which in the end resulted in stimulation of vascular remodeling such as angiogenesis [92]. A further study on synergistic effects of GF in angiogenesis in a mouse corneal micropocket assay showed that PDGF-BB with FGF-2 had a positive effect, whereas for other combinations with VEGF, this could not be shown, even though all three are known as angiogenic factors [93]. For IGF-1 in combination with VEGF-induced survival, proliferation and secondary sprouting were observed for retinal endothelial cells, whereas they showed a decreased synergistic effect in a wound migration assay [94]. This interplay suggests that a coordinated administration of different growth factors could be beneficial in therapeutic angiogenesis [90]. As we could see an increase of especially junction count and vessel density stimulated by both mixed groups, we can assume a combinatorial effect promoted by applying both GFs. As the different concentrations had slightly different results, it is important to further analyze the effects triggered by different concentrations or ratios of GF application. Also, the vessel hierarchy was altered over time, especially when GFs were applied in combination, and the count of smaller vessels outperformed that of lager vessels, particularly at later timepoints, as also indicated by the larger vessel density on d7. In conclusion, the CAM assay serves as a valuable platform for studying angiogenesis and the effects of various growth factors. The roles of PDGF-BB and IGF-1 in promoting and stabilizing new blood vessel formation highlight their potential in clinical applications aimed at enhancing vascularization [95].

Transection reduced biomechanical properties, as evidenced by reductions in failure stress, stiffness, and elastic modulus across all treated groups compared to non-treated tendons. Notably, the GF tubes demonstrated slightly better mechanical properties than the control tube, suggesting that the integration of either PDGF-BB alone or in combination with IGF-1 supports superior tissue integration and functional recovery. As PDGF-BB is known to enhance cell proliferation and collagen deposition in the early stages of healing [14], the superior function of the GF tube can be explained. With IGF-1 [61], we suggest that these effects could be further enhanced.

The observed increase in CSA in treated tendons likely reflects swelling, tissue remodeling, or scar formation, common during the early remodeling phase of tendon healing [1]. At this stage, while tendons are typically functional, the alignment and maturation of collagen fibers are still ongoing, which may contribute to the observed alterations in mechanical properties. Despite these structural changes, mechanical strength often does not fully recover to pre-injury levels, as factors such as collagen organization and cross-linking are still maturing [25]. This is consistent with previous studies showing that these tubes not only reduce adhesion formation by approximately 20% [66] but also significantly enhance the biomechanical properties of tendons three weeks after surgery [22]. A further study demonstrates that, even at six weeks post-treatment with TGF-β3, tendons exhibited improved but not fully restored mechanical properties compared to intact tendons [96].

The same mechanical performance of the mixed tube and the PDGF-BB tube suggests that the combined approach may not provide an additive benefit in biomechanical recovery at this time point.

The results from adhesion experiments confirmed trends observed from biomechanics. The NT group exhibited the lowest adhesion levels, representing an unaltered tendon environment, which confirms that natural tendon surfaces maintain minimal interaction with outer tissues when not disturbed. The four-strand group, where tendons were cut and sutured, demonstrated the highest adhesion levels. This suggests that surgical intervention and subsequent healing lead to increased fibrotic interactions between the tendon and surrounding tissues, a phenomenon well-documented in tendon healing studies [97,98].

The groups which also involved sutured tendons with an additional bioactive tube applied over them showed intermediate adhesion levels compared to the NT and were significantly lower than the four-strand groups. This indicates that the tubes provided a partial barrier, reducing but not completely preventing adhesion formation. Previous research has shown that 3D printed sleeves around injured tendons can help modulate healing by limiting excessive scar tissue formation while still allowing sufficient integration for functional recovery [99]. The differences between the control tube and mixed tube groups could be attributed to variations in tube composition, mechanical properties, or bioactivity.

Although significant differences in proliferation between IGF-1 and both GFs on day 7 were observed, with further significant differences in the CAM assay, the cell morphology (aspect ratio) and a Warburg-like shift in the Seahorse experiments under PDGF-BB compared to both GFs and the in vivo outcome with both GFs applied simultaneously did not reflect this impact. We conclude that either longer periods than a 3-week experiment or higher concentrations of GFs released to the healing tendon are necessary to observe a clear impact of IGF-1 released when PDGF-BB is released, which encourages further research behind the synergistic effect of IGF-1 and PDG-BB.

## 4. Materials and Methods

### 4.1. Synthesis of DegraPol^®^ (DP)

To synthesize DP, a mixture consisting of 25 wt% poly(3-(R-hydroxybutyrate)-co-(ε-caprolactone)-diol (Mn = 2824 g/mol)) and 75 wt% poly(ε-caprolactone)-diol-co-glycolide (15 mol% glycolide, 85 mol% ε-caprolactone) (Mn = 1000 g/mol) was dissolved in 1,4-dioxane and dried until the water content was reduced to below 20 ppm. The resulting solution was cooled, and a stoichiometric amount of 2,2,4-trimethylhexane-diisocyanate (TMDI) was added. After one day, dibutyltin dilaurate (20 ppm) was incorporated three times within the day to reach a molecular weight range of 100–110 kDa. The polymer was precipitated in cooled hexane isomers, purified using chloroform and a silicagel 60 column (Fluka, Charlotte, NC, USA), and further purified through precipitation in cooled ethanol. The batch was produced in September 2022.

### 4.2. Scaffold Production and Incorporation of GFs

Polymer solutions were prepared at least one day prior to electrospinning to ensure complete dissolution and stability. For each scaffold, a PEG (35 kDa, Aldrich 81310) solution was made by dissolving 1.5 g of PEG in 3.5 g of chloroform (Sigma-Aldrich 132950). The DP solution was formulated separately by mixing 0.6 g of DP powder with 3.52 g of chloroform and 0.88 g of 1,1,1,3,3,3-Hexa Fluoro-2-Propanol (HFP, Aldrich 105228) in a screw-cap glass container. To incorporate GFs, either 8 µg of PDGF-BB or 4 µg of IGF-1—dissolved in 200 µL of phosphate-buffered saline (PBS) containing RSA—was added dropwise into the DP solution under continuous stirring at 500 rpm for five minutes using a magnetic stirrer. The resulting mixture was briefly vortexed and subsequently emulsified in an ultrasonic bath for 15 min to ensure proper dispersion. The final emulsion was transferred into a 5 mL glass syringe (Huberlab via P4U UZH Irchel, 3.7102.33, Industriestrasse 123, Aesch, Switzerland) and used immediately for electrospinning.

Tubular scaffolds were produced using a custom-designed electrospinning setup that included a DC high-voltage power supply (Glassman High Voltage Inc., High Bridge, NJ, USA), a syringe pump (SP210cZ, WPI, Friedberg (Hessen), Germany), and a needle holder mounted on a lateral transporter. The polymer solution was conveyed through a Teflon hose into a blunt-ended stainless steel needle (1 mm inner diameter, 0.3 mm wall thickness; Angst & Pfister AG, Zürich, Switzerland). This needle dispensed the solution onto 550 mm-long metal rods, which were attached to a rotating motor (Euro Star B, IKA Labortechnik, Staufen, Germany) acting as the fiber collector. Electrospinning was performed at room temperature (22–23 °C) and under controlled humidity conditions (25–35%). The parameters for electrospinning included a flow rate of 1 mL/h, a 19.5 cm distance between the needle tip and the collector, and an applied voltage of 12.5 kV. To ensure uniform fiber deposition, the needle was programmed to move laterally across a 20 cm range, while the collector rotated at 500 rpm. To enable easy scaffold removal, a preliminary layer of PEG was electrospun onto the surface of the metal rod. Once the base layer was established, additional layers consisting of either DP alone or DP blended with GFs were deposited. For scaffold retrieval, 50% ethanol was pipetted directly onto the tubular structures, and the scaffolds were gently released from the collector using tweezers. Following detachment, they were immersed in 50% ethanol, rinsed thoroughly with distilled water, and dried in a desiccator. The drying period ranged from three to seven days, after which the scaffolds were stored at 4 °C until use.

### 4.3. SEM Fiber and Pore Size Analysis

Small sections from each scaffold tube, including both inner and outer surfaces, were prepared for imaging. The samples were affixed to SEM stubs using conductive double-sided adhesive tape. All coating and imaging procedures were conducted using equipment maintained by the Center for Microscopy and Image Analysis at the University of Zurich. Prior to imaging, the samples were coated with a 10 nm layer of platinum using a Safematic CCU-010 sputter coater. SEM was then performed with a Zeiss Gemini SEM 450 operating at an accelerating voltage of 5 kV. Images were captured at 500× magnification using the secondary electron detector with a brightness setting of 49%. Fiber diameters and tube wall thicknesses were quantified using ImageJ software (version 1.53e/Java 1.8.0_172, 64-bit). Measurements were based on the scale bars provided in the SEM images. For analysis, a diagonal reference line was drawn across each image, and all fibers or pores intersecting this line were measured.

### 4.4. WCA Evaluation

The WCA was assessed for both pure DP tubes and DP emulsion tubes containing GFs. To prepare the samples, each tube was carefully opened lengthwise using a scalpel and mounted flat onto a glass plate using double-sided adhesive tape to secure both the inner and outer surfaces for measurement. A goniometer equipped with an IDS uEye camera was used to capture the contact angle. Milli-Q water droplets (5 µL each) were dispensed onto the sample surface using a 1 mL syringe. For each droplet, the left and right contact angles were recorded, and their average was used to determine the static WCA. A minimum of three measurements was performed for each sample to ensure accuracy.

Dynamic WCA measurements were also conducted using the same goniometer setup. Starting with a 5 µL droplet, water was either added or withdrawn at a controlled rate of 15 µL/min to determine the advancing and receding contact angles, respectively. Measurements were taken at one-second intervals over the course of one minute. WCA hysteresis was calculated as the difference between the advancing and receding angles.

### 4.5. FTIR Analysis

FTIR spectroscopy was carried out using a Varian 640 FTIR spectrometer equipped with a Golden Gate diamond ATR unit featuring temperature control. Spectra were collected over a wavenumber range of 600 to 4000 cm^−1^ at a resolution of 4 cm^−1^. Each spectrum represented an average of 64 individual scans to enhance signal quality. For comparative evaluation, the intensity ratio of the C=O absorption peak at 1720 cm^−1^ to the C–O peak at 1175 cm^−1^ was determined. All spectra were normalized relative to the C=O peak at 1720 cm^−1^. The analysis included DP powder, unmodified DP tubes, and DP tubes containing different emulsions. PEG was also examined separately to assess possible contamination. To identify characteristic functional groups, the recorded peaks were compared against values listed in an IR reference spectrum table (Merck KGaA, Darmstadt, Germany).

### 4.6. Release and Lipase Assay

To evaluate the absorption and release of GFs from the DP scaffolds, three 0.5 cm-long segments were taken from each tube, one from each end and one from the center, and individually placed into low-binding microtubes (Eppendorf, Schönenbuch, Switzerland). Each segment was immersed in 500 µL of 0.1% RSA in PBS, which served as the release medium. The samples were incubated at 37 °C with constant shaking at 300 rpm. At predefined time points, the release medium was carefully collected into labeled microtubes, and an equal volume of fresh medium was added to the original tubes to continue the release process. All collected samples were stored at −20 °C for subsequent analysis.

Quantification of GF release was performed using either the Human IGF-1 ELISA Kit or the Human PDGF-BB ELISA Kit (both from Peprotech, Cranbury, NJ, USA) in accordance with the manufacturer’s protocols. Absorbance readings were obtained using a BioTek Cytation/5 imaging microplate reader (Im Kirschgarten 30, Schönenbuch, Switzerland), with measurements taken at 450 nm and corrected at 630 nm. Results were reported as cumulative release values over time (ng/mL). Following the final incubation time point, the scaffolds were stored in the residual PBS at −20 °C for preservation.

To evaluate the initial GF loading in DP scaffolds, which were fabricated using electrospinning, the scaffolds were enzymatically degraded, and the released GFs were quantified. Lipase from Thermomyces lanuginosus (Sigma-Aldrich, St. Louis, MO, USA), an esterase, was used to hydrolyze the ester bonds in DP, allowing scaffold degradation and GF recovery.

The scaffolds, previously used to measure release kinetics, were placed in low-protein-binding microtubes (Eppendorf, Switzerland), and 1 mL of lipase solution (10,000 U/mL) in a serum-free culture medium was added. The medium consisted of Ham’s F12 supplemented with 1× RPMI vitamin solution (Sigma-Aldrich, London, UK), 1× non-essential amino acids (Life Technologies, Paisley, UK), and 200 µg/mL gentamicin. The samples were incubated at 37 °C and 5% CO_2_ with gentle shaking for three days. During the incubation, periodic vortexing was performed, and after incubation, the scaffolds were further disintegrated using a pipette tip to maximize scaffold degradation and GF release.

After disintegration, the samples were centrifuged at 10,000 rcf for 15 min. The supernatant containing the released GFs was collected and stored at −20 °C for subsequent analysis. GF quantification was performed using the DC Protein Assay (Bio-Rad, Hercules, CA, USA, Nr. 5000111) according to the manufacturer’s protocol. Measurements were conducted using a Microplate Reader (BioTek Cytation/5 imaging reader) at 450 nm.

### 4.7. Cell Culture (Including Image Capture, Aspect Ratio, AB, and qPCR)

Cell Culture: RbTenocytes isolated from the Achilles tendons of New Zealand White rabbits and rbASCs were utilized in this study. After thawing, the cells were resuspended in a culture medium composed of Ham’s F12 for tenocytes and DMEM (Thermo Fisher Scientific, Zurich, Switzerland) for ASCs, each supplemented with 10% fetal bovine serum (FBS), 1% penicillin/streptomycin (P/S) (Life Technologies), and 1% Glutamax (Thermo Fisher Scientific, Switzerland). Tenocytes at passage 2 and ASCs at passages 3–4 were used for experiments. The effect of GFs on tenocytes was assessed using the alamarBlue™ and qPCR assays in culture medium.

Cell Proliferation: Cell proliferation was assessed on culture days 0, 1, 3, 7, and 14 using the alamarBlue™ cell viability assay (Thermo Fisher Scientific, Waltham, MA, USA). For each time point, 350 cells were seeded per well in a 96-well plate (TPP, #92096), with a total volume of 100 µL culture medium per well. Technical quadruplicates were used for each condition. Plates were incubated at 37 °C in a humidified incubator with 5% CO_2_. To minimize evaporation, surrounding empty wells were filled with PBS.

The alamarBlue™ reagent was diluted 1:10 in fresh culture medium and added to the wells. After a 4-h incubation period, fluorescence was measured using a BioTek Cytation/5 imaging reader, with excitation and emission wavelengths set at 530 nm and 590 nm, respectively.

Real-time PCR: To examine the effects of GFs on rbTenocyte gene expression over time, cells were cultured and processed accordingly. Tenocytes were seeded into 6-well plates (Sigma, #SIAL0516; growth area: 9.6 cm^2^ per well) at a density of 2 × 10^5^ cells per well in 2 mL of standard culture medium. After allowing cells to adhere overnight, GFs were added at the desired concentrations on day 0. Samples were harvested after 3 and 7 days of incubation. Cells from three independent donors were used, and quantitative PCR (qPCR) reactions were conducted in technical triplicates.

Total RNA was extracted using the RNeasy Plus Mini Kit (Qiagen, Hilden, Germany), which included an RNase-free DNase digestion step (Qiagen, #74104) in accordance with the manufacturer’s instructions. RNA concentration and purity were assessed using a Nanodrop One spectrophotometer (Thermo Fisher Scientific). For reverse transcription, 500 ng of total RNA was converted into cDNA in a 20 µL reaction volume. The RT reaction was carried out using SuperScript III Reverse Transcriptase (Thermo Fisher, #18080085), Oligo(dT)12–18 primers (Thermo Fisher, #18418012), RNase inhibitor (Applied Biosystems, Waltham, MA, USA, #N8080119), and dNTPs using a Mastercycler Personal thermocycler (Eppendorf).

QPCR was performed on a QuantStudio 5 system (Applied Biosystems) using Fast SYBR Green Master Mix (Thermo Fisher, #4385612). Each reaction was run in technical triplicate under the following cycling conditions: initial denaturation at 95 °C for 3 min followed by 40 cycles of 95 °C for 3 s and 60 °C for 20 s. Primer sequences (listed in Table 1 were synthesized by Microsynth (Balgach, Switzerland).

Relative gene expression levels were calculated using the 2^−ΔΔCT^ method. All expression values were normalized to the 18S rRNA gene, which remained stable under all experimental conditions. Final results were expressed as fold changes relative to control samples (no GF treatment), which were set to a baseline value of 1.

### 4.8. Cell Seeding on Different Scaffolds

To investigate cell adhesion, four different scaffold types as were used: one control and three groups with individual or mixed GF incorporation. Circular samples with a diameter of 5 mm were first punched out from each scaffold type. The individual samples were then sterilized under UV light for 30 min on each side. To improve surface wettability, the scaffolds were briefly immersed in 50% ethanol, rinsed three times with sterile water, and washed once with a cell culture medium.

The pre-treated samples were placed into a 12-well plate. RbTenocytes (passage 2) were seeded directly onto the surface of the scaffolds at a density of 1 × 10^5^ cells in 10 µL of medium. Cells were allowed to attach for approximately 3 h at 37 °C and 5% CO_2_ before 1 mL of complete culture medium was added to each well. The medium was changed every three days.

Separate samples were prepared for each time point (days 3, 7, and 14). After each respective culture period, scaffolds were fixed in a solution of either 0.5% glutaraldehyde or 3% PFA for SEM preparation and stored at 4 °C.

For SEM, rbTenocyte-seeded scaffolds were first rinsed once with PBS to remove residual medium. Dehydration was carried out through a graded ethanol series with increasing concentrations of 30%, 50%, 70%, 80%, and 95% ethanol (each for 5 min) followed by two washes in 100% ethanol for 10 min each.

Following dehydration, chemical drying was performed using a graded hexamethyldisilazane (HMDS)/ethanol mixture in the following ratios, 1:3, 1:1, and 3:1, each for 15 min. This was followed by incubation in pure HMDS for an additional 15 min. The HMDS was then allowed to evaporate overnight under a fume hood. Finally, the dried scaffolds were mounted onto SEM stubs for imaging as described above.

### 4.9. Seahorse

OCR and ECAR were assessed in cells using a Seahorse XF-96 analyzer. Rabbit tenocytes and ASCs were seeded at 7500 cells per well in 80 μL of growth medium in Seahorse microplates and incubated at 37 °C in 5% CO_2_ for 24 h. For the assays, the growth medium was replaced with 200 μL of pre-warmed XF RPMI medium (pH 7.4, Agilent Technologies, 103576-100) containing 100 mM glucose, 1 mM pyruvate, and L-Glutamine for the mito stress test or without glucose for the glycolysis stress test. The cells were then incubated at 37 °C for 60 min to allow the medium temperature and pH to stabilize. Once equilibrated, OCR (in pMoles/min) was measured as an indicator of mitochondrial respiration, and ECAR (in mpH/min) was measured as an indicator of lactate production or glycolysis.

Following baseline measurements, 20, 22, and 25 μL of reagents prepared in an assay medium were injected into each well to achieve the final concentrations as follows: Port A with 1.5 μM oligomycin, Port B with 6 μM FCCP, and Port C with 0.5 μM rotenone and antimycin for the cell mito stress test; Port A with 10 μM Glucose, Port B with 1 μM oligomycin, and Port C with 50 μM 2-deoxy-glucose (2-DG) for the glucose stress test. New OCR and ECAR values were recorded after each injection. Baseline and response rates were measured three times, and the average of three readings from each phase was used for analysis. At the end of the assay, the cells were stained with Höchst (Thermo Fischer 33342), and cell density was measured by the Agilent BioTek Cytation 1 cell imaging multimode reader. The key parameters included basal respiration, maximal respiratory capacity, basal acidification rate, and maximum acidification rate and were analyzed by Agilent Seahorse Analytics (Version 1.0.0-739, Agilent Technologies, Santa Clara, CA, USA). Graphical content and statistics were performed in GraphPad Prism 10 (Version 10.3.1, GraphPad Software Inc., San Diego, CA, USA).

### 4.10. CAM

In accordance with Swiss animal care guidelines (TSchV, Art. 112), experiments involving chicken embryos up to embryonic day 14 do not require IACUC approval. Fertilized Lowman White LSL chicken eggs (Animalco AG Geflügelzucht, Staufen, Switzerland) were incubated at 37 °C with 65% relative humidity for 4 days. On embryonic day 4, 5 mL of albumen was removed to facilitate detachment of the developing chorioallantoic membrane (CAM) from the eggshell. A circular window was then carefully excised in the eggshell and incubated for 3 further days until embryonic development day 7 (EDD7).

GF mixtures were prepared by diluting the respective GFs in PBS to achieve the desired final concentrations. The experimental groups included the control, 50 ng/mL IGF-1, 50 ng/mL PDGF-BB, a combination of 50 ng/mL IGF-1 + 50 ng/mL PDGF-BB, 1 ng/mL IGF-1, 25 ng/mL PDGF-BB, and a combination of 1 ng/mL IGF-1 + 25 ng/mL PDGF-BB.

On days 0 (EDD7), 2 (EDD9), 4 (EDD11), and 6 (EDD13) of the experiment, 50 µL of the respective GF solution was applied in the center of a 0.8 cm diameter silicon ring placed on the CAM surface. This served not only to deliver the GFs but also to flatten the CAM and create a clear landmark for subsequent tracking of the treated area. On days 0 (EDD7), 2 (EDD9), 4 (EDD11), and 7 (EDD14), pictures were taken using a Zeiss Axio Vert.A1 brightfield microscope with ZEN 2.6 lite software (Carl Zeiss Microscopy, Oberkochen, Germany). At the end of the experiment, the CAM was excised and prepared for further histological analysis. Parameters were assessed manually with the help of the ImageJ software (version 2.9.0, NIH, Bethesda, MD, USA).

### 4.11. In Vivo Experiments: Biomechanics and Adhesion Extent

To evaluate the implantation of GF tubes, three female New Zealand White rabbits (ages 12 to 16 weeks, specific pathogen free—SPF) were used per group. These animals, sourced from Charles River (Research Models and Services, Germany), were housed, maintained, and fed as described in a previous study [100]. A two-week acclimatization period preceded any surgical procedures. Approval for the animal experiments was granted by the Zurich veterinary authority (reference numbers: ZH 080/2021; 33530). Surgical intervention involved a complete transection of the Achilles tendon, performed 2 cm proximal to the calcaneus, followed by repair using a 4-strand Becker suture based on established protocols [100].

Prior to implantation, the tubes underwent plasma sterilization using hydrogen peroxide. During surgery, the tubes were flipped inside-out to ensure the smooth layer out of pure DP faced the surrounding tissue. In contrast, the rough outer layer, containing PDGF-BB, was positioned adjacent to the sutured tendon. Closure of the surgical site was performed with a continuous suture using USP 6.0 polypropylene fiber. Post-surgery, a well-padded cast was applied with the ankle set at a 180° angle. For analgesia, each rabbit received a Durogesic Matrix patch (Janssen-Cilag AG, Zug, Switzerland) containing 4.2 mg of Fentanyl, delivering 25 μg/h over approximately 72 h.

Three weeks post-operation, animals were euthanized under deep anesthesia (administered with 100 mg/kg Ketamine and 4 mg/kg Xylazine) followed by an overdose of 80 mg/kg Pentobarbital (Esconarkon ad us. vet., Switzerland). Only one hind limb underwent surgery, while the contralateral limb remained untreated (NT), serving as a control. Extracted tendons were promptly frozen at −20 °C in gauze moistened with a 0.9% NaCl solution.

Prior to mechanical testing at room temperature (21 °C), the tendons were thawed overnight at 4 °C. Each tendon was harvested from the hind limb, including both the muscle and the calcaneus (sutures were not removed). For mounting, the muscle end of the sample was wrapped in two layers of cloth to minimize slippage and then secured in serrated clamps, following the method described by Rigozzi et al. (2009) [101]. On the bone side, a custom device was used to hold the calcaneus in a fixed rectangular position, ensuring a 90-degree angle between the calcaneus and the longitudinal axis of the stretched tendon. All samples underwent uniaxial tensile testing to failure at a constant speed of 1 mm/min using a universal testing machine (Zwick 1456, equipped with a 1 kN load cell, TestXpert 10, Germany). Before the failure test, tendons were preconditioned with 10 loading cycles up to 10 N. To prevent dehydration during testing, samples were continuously moistened with PBS. The maximum load recorded during testing was taken as the load to failure (N). Prior to tensile testing, the cross-sectional area (CSA) was measured 2.0 cm proximal to the calcaneus using a custom-designed linear laser scanner adapted from the method of Vergari et al. (2010) [102] and Fessel et al. (2014) [103], with six measurements taken per specimen (*n* = 6). Failure stress at the repair site (MPa) was then calculated by dividing the load to failure by the CSA (in mm^2^). Finally, the elastic modulus (E-Modulus; MPa) was determined as the slope of the linear region of the stress–strain curve.

After mechanical testing, tendons were immediately prepared for histological analysis by dehydration and embedding in paraffin following standard procedures. Cross-sections of 5 μm, cut perpendicular to the tendon axis at the injury site, were deparaffinized using xylene, rehydrated, and stained with Hematoxylin–Eosin (H&E), Alcian Blue (AB), Hemalaun Sudan (HS), and Picrosirius Red (PS) using established staining methods.

To assess the degree of tissue adhesion, five consecutive PS-stained cross-sections spaced 2.0 mm apart were evaluated under 8× magnification using a Leica EZ4D microscope (Switzerland) following the method outlined by Tan et al. (2010) [104]. Adhesion was quantified by measuring both the tendon’s contact length with surrounding tissue and its total perimeter. The ratio of these two measurements, calculated using Synedra View software (version 22.0.0.12), represented the percentage of adhesion.

### 4.12. Statistics

Data analysis was performed using GraphPad Prism 10 (Version 10.3.1, GraphPad Software Inc., San Diego, CA, USA). Normality of the data was assessed using the Shapiro–Wilk test. For comparisons involving more than two groups, a one-way analysis of variance (ANOVA) with Tukey’s multiple comparisons test was performed if the data were normally distributed. If the data did not follow a normal distribution, a nonparametric Kruskal–Wallis test was employed. A two-way ANOVA was used if more than one parameter was compared. A *p*-value of ≤0.05 was considered statistically significant and indicated with (*), *p* ≤ 0.01 (**) was used for greater significance, *p* ≤ 0.001 (***) for very high significance, and *p* ≤ 0.0001 (****) for extremely high significance, with (ns) indicating no significance. Standard curve calculations and other relevant analyses were conducted using Excel (Version 2402 Build 16.0.17328.20550, 64-bit).

## 5. Conclusions

The results of this study highlight the potential of IGF-1 and PDGF-BB as bioactive components for tendon regeneration. Scaffolds containing IGF-1 enhanced tenocyte proliferation and mitochondrial activity, while the combination of IGF-1 and PDGF-BB exhibited partially synergistic effects, particularly in terms of cellular metabolism. Notably, all GF-containing scaffolds showed comparable structural properties and improved hydrophilicity due to the incorporation of aqueous GF solutions during fabrication. The CAM assay further revealed a slight trend toward increased angiogenesis in the combination group compared to individual GF treatments. In vivo, the combination scaffold performed equally well in biomechanical strength and adhesion formation as the single-factor groups and was significantly superior to the untreated control.

Importantly, the combined application of IGF-1 and PDGF-BB did not result in a simple additive effect but rather induced a complex and time-dependent biological response. Some parameters were enhanced in single-factor treatments at early time points, whereas the combination led to stronger effects later on. This indicates a synergistic modulation of cellular and molecular processes—both stimulatory and inhibitory—depending on context.

## Figures and Tables

**Table 1:** Primer sequence genes from which expressions were measured, the NCBI reference sequence (used as template for primer design), amplicon size, and forward and reverse primer sequences used for qPCR analysis.

Gene	mRNA ID (NCBI Reference Sequence)	Amplicon Size (bp)	Forward Primer Sequence (3′ → 5′)	Reverse Primer Sequence (5′ → 3′)
**18s rRNA**	NR_033238.1	176	GGAACTGAGGCCATGATTAAG	CGGAACTACGACGGTATCTG
**Col1A1**	XM_008271783.1	271	CTGGTGAATCTGGACGTGAG	TGTCTCACCCTTGTCACCAC
**Tenomodulin**	NM_001109818.1	239	GCAGTTTCCGAGTTACAAGAC	CGACGGCAGTAAATACAACAG
**Ki67**	XM_008251084.2	283	CACATCCAGCAGTGAAACGG	GTGTTAGCAGTACCTGAAGTC
**MTROC 1**	XM_088251668.2	117	GTTTGTCGCGGACGTTTAAG	AGGAAAGGCATGACAAAGGC

## Data Availability

Data are contained within the article and Supplementary Materials.

## Supplementary Materials

The following supporting information can be downloaded at: https://www.mdpi.com/article/10.3390/ijms26094039/s1.
