# Supplementary material for: Synergistic Effects of Insulin-like Growth Factor-1 and Platelet-Derived Growth Factor-BB in Tendon Healing
Source: Int J Mol Sci. 2025 Apr 24;26(9):4039. doi: 10.3390/ijms26094039 (PMC12072114; doi:10.3390/ijms26094039)
Supplement: Supplementary file 1 [file ijms-26-04039-s001.zip › ijms-3589237-supplementary.pdf]

International Journal of
Molecular Sciences

MDPI

Supplementary Materials

Synergistic Effects of Insulin-like Growth Factor-1 and Platelet-Derived Growth Factor-BB in Tendon Healing

Julia Rieber [1], Petra Wolint [1], Gabriella Meier-Bürgisser [1], Esteban Ongini [2,3], Pietro Giovanoli [1], Maurizio Calcagni [1], Jess G. Snedeker [2,3] and Johanna Buschmann [1,*]

[1] Division of Plastic Surgery and Hand Surgery, University Hospital Zurich, Sternwartstrasse 14, 8091 Zurich, Switzerland; julia.rieber@usz.ch (J.R.); petra.wolint@usz.ch (P.W.); gabriella.meierbuergisser@usz.ch (G.M.-B.); pietro.giovanoli@usz.ch (P.G.); maurizio.calcagni@usz.ch (M.C.)
[2] Institute for Biomechanics, ETH Zurich, 8092 Zurich, Switzerland; esteban.ongini@hest.ethz.ch (E.O.); jess.snedeker@hest.ethz.ch (J.G.S.)
[3] Balgrist University Hospital, University of Zurich, 8008 Zurich, Switzerland
* Correspondence: johanna.buschmann@usz.ch; Tel.: +41-44-255-98-95

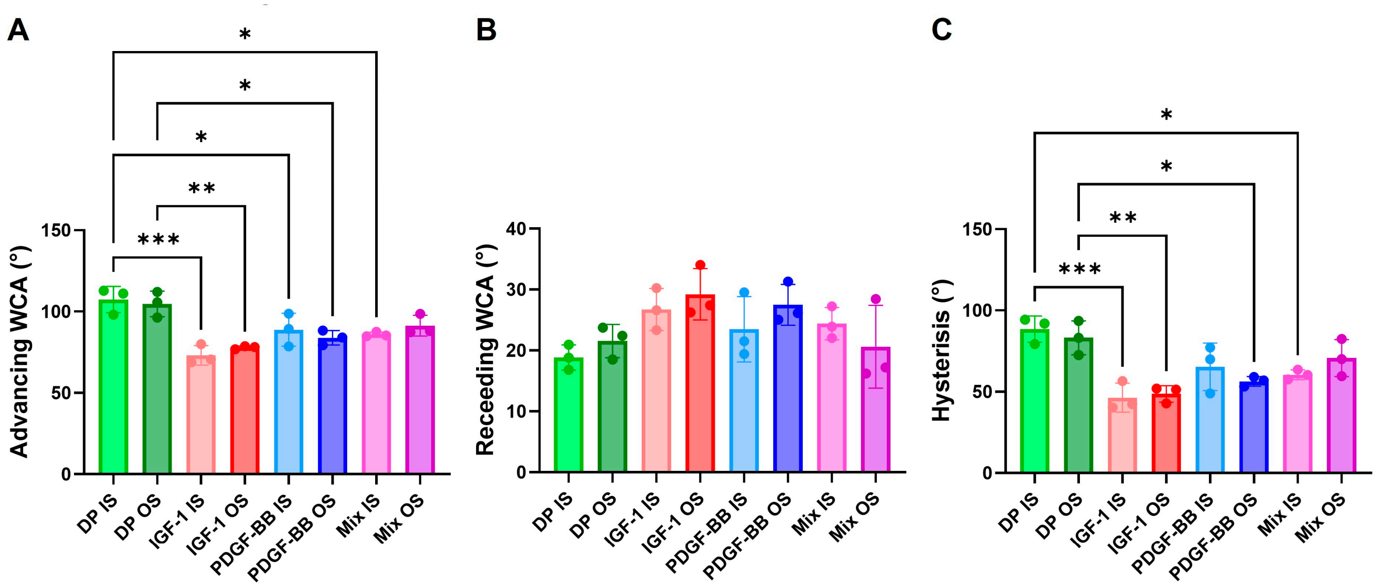

Figure S1. Statistical analysis of dynamic water contact angel The advancing (**A**) receding (**B**) water contact angle and hysteresis (**C**) were analyzed statistically for the 4 groups on each inner and outer surface. Data is shown as mean and SD, n = 3. Normality was assessed using the Shapiro-Wilk test. As data were normally distributed, group comparisons were performed using one-way ANOVA followed by Tukey's multiple comparisons test. P-values ≤ 0.05 were considered significant and denoted as: $p \leq 0.05$ (*); $p \leq 0.01$ (**); $p \leq 0.001$ (***).

 https://doi.org/10.3390/ijms26094039

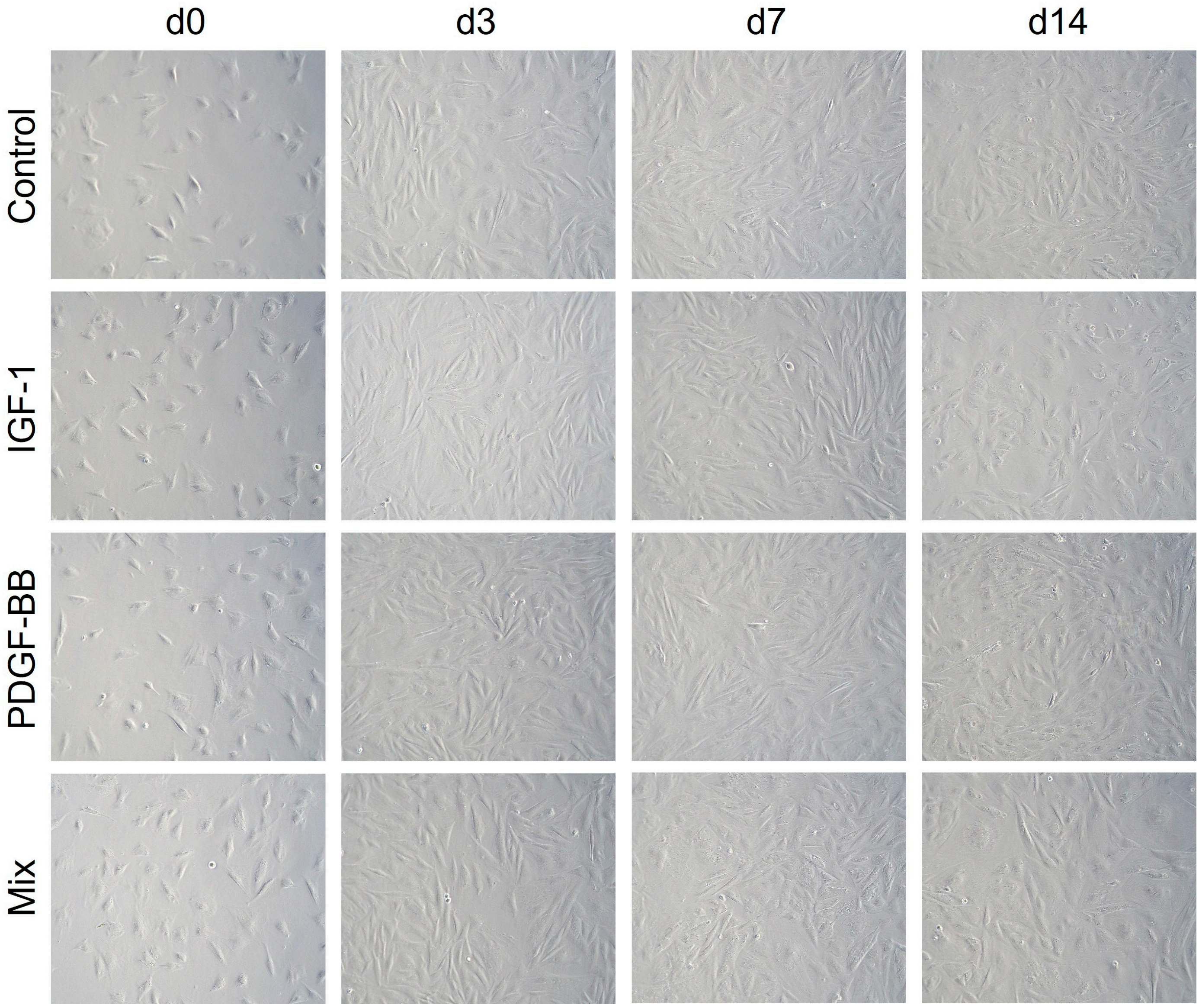

Figure S2. Microscopic images of rbTenocytes cell culture with different treatments (control, 1ng/mL IGF-1, 25ng/mL PDGF-BB or mix of both at same concentration). Images were assessed at d0, d3, d7 and d14.
